# Intercomparison of Radon Flux Monitors at Low and at High Radium Content Areas under Field Conditions

**DOI:** 10.3390/ijerph19074213

**Published:** 2022-04-01

**Authors:** Daniel Rábago, Luis Quindós, Arturo Vargas, Carlos Sainz, Ileana Radulescu, Mihail-Razvan Ioan, Francesco Cardellini, Marco Capogni, Alessandro Rizzo, Santiago Celaya, Ismael Fuente, Marta Fuente, Maria Rodriguez, Claudia Grossi

**Affiliations:** 1 Radon Group, University of Cantabria, 39011 Santander, Spain; daniel.rabago@unican.es (D.R.); carlos.sainz@unican.es (C.S.); santiago.celaya@unican.es (S.C.); fuentei@unican.es (I.F.); 2 Laboratory of 222Rn Studies, Institut de Tècniques Energètiques, Universitat Politècnica de Catalunya, 08028 Barcelona, Spain; arturo.vargas@upc.edu (A.V.); maria.dolores.rodriguez@upc.edu (M.R.); claudia.grossi@upc.edu (C.G.); 3 Horia Hulubei National Institute for R&D in Physics and Nuclear Engineering, 077125 Magurele, Romania; rileana@nipne.ro (I.R.); razvan.ioan@nipne.ro (M.-R.I.); 4 National Institute of Ionizing Radiation Metrology (INMRI)—Italian National Agency for New Technologies, Energy and Sustainable Economic Development (ENEA), Via Anguillarese 301, 00123 Rome, Italy; francesco.cardellini@enea.it (F.C.); marco.capogni@enea.it (M.C.); 5 Radiation Protection Institute (IRP)—Italian National Agency for New Technologies, Energy and Sustainable Economic Development (ENEA), Via Anguillarese 301, 00123 Rome, Italy; alessandro.rizzo@enea.it; 6 Laboratoire des Sciences du Climat et de l’Environnement, (LSCE-IPSL), CEA-CNRS-UVSQ, Université Paris-Saclay, 91191 Gif-sur-Yvette, France; marta.fuente-lastra@lsce.ipsl.fr

**Keywords:** exhalation, traceRadon, proficiency test, interlaboratory comparison

## Abstract

Interlaboratory exercises are a good tool to compare the response of different systems to the same quantity and to identify possible inconsistencies between them. One of the main goals of the EMPIR 19ENV01 traceRadon project is to harmonize radon flux measurements based on different systems and methodologies. In the framework of the traceRadon Project, two radon flux intercomparison campaigns were carried out in October 2021 at high and at low radon source areas. Four institutions participated in the field intercomparison exercises with their own systems. Every system was based on a specific radon monitor (diffusion or pump mode) and an accumulation chamber (with manual or automatic opening). Radon fluxes were calculated by each participant using both exponential and linear fittings of the radon activity concentration measured over time within the accumulation chambers. The results of this study show mainly: (i) the exponential approach is not advisable due to the variability of the radon flux and the leakage of the systems during long-time measurements; (ii) the linear approach should be applied to minimize the measurement period in agreement with the time response and sensitivity of the monitors; (iii) radon flux measured at high radon source areas (radium content of about 800 Bq kg^−1^) risks being underestimated because of the influence of advective effects; (iv) radon flux measured at low radon source areas (radium content of about 30 Bq kg^−1^) may present large uncertainties if sensitive radon monitors with pump mode are not used.

## 1. Introduction

The measurement of radon exhalation rate from the ground, also called radon flux, represents the radon activity concentration that escapes from the soil per unit of surface and unit of time. It is usually expressed in Bq m^−2^ s^−1^ or Bq m^−2^ h^−1^. Radon fluxes are commonly used to determine the radon potential of an area, helping to identify the areas that need regulatory requirements [1]. Currently, the European Council Directive 2013/59/EURATOM (EU-BSS) [2] requires all EU member states to develop a Radon Action Plan, which should include the identification of Radon Priority Areas (RPA) where the annual average radon concentration in buildings is expected to exceed the national reference level [3]. In addition, radon flux observations are also useful for climate research goals. For example, the Radon Tracer Method (RTM) [4] uses known radon fluxes and co-located atmospheric measurements of radon activity concentration and greenhouse gases (GHGs) over areas to estimate GHGs emissions, which is needed to evaluate reduction strategies [5]. In that framework, properly validated radon flux maps can assist reaching both climate and radiation protection aims. The validation of radon flux maps, based both on static inventories [6] and dynamic models [7], requires high-quality radon flux observations. The traceRadon project [8] aims to provide a metrological chain for radon flux measurements for their use in the RTM, for GHG flux retrieval, and in the identification of RPA [9].

The first radon exhalation rate studies had a theoretical approach, in which assumptions over diffusion were made and the resultant equation solved [10,11,12,13,14]. This was completed using a thin layer model and a soil with well-characterized ^226^Ra concentration, depth (thickness), porosity, and radon emanation characteristics. Other studies were experimentally based on the accumulation chamber method [15,16], resulting as a standard method reflected in the ISO 11665-7:2012 “Accumulation method for estimating surface exhalation rate” [17]. However, the quality and reliability of radon flux measurements is still challenging because of the influence that the monitor response, the installation of the accumulation chamber, the environmental parameters, and the soil properties could have on the measurements themselves, as reported by [18,19]. Research in this field is still ongoing, and many questions need to be answered.

The measurement of radon fluxes in situ can be performed by coupling continuous radon monitors with accumulation chambers installed on the soil surface. Accumulation chambers can be automatically controlled to open/close to allow the reduction/increase in radon activity concentration inside them for every measurement. A literature review conducted within the traceRadon project showed that there are several radon flux systems available with different accumulation chamber volumes, shapes, and characteristics. Still, there is no harmonization between the existing radon flux systems and methods. In addition, there is a lack of interlaboratory exercises to compare such systems and to identify possible inconsistencies between their responses [20].

To fulfill the previous need, two intercomparison campaigns were conducted in northwestern Spain at low and at high radium content areas in the EMPIR 19ENV01 traceRadon project framework. The main goal of the experiments was to test the response of radon flux systems based on different monitors and different accumulation chambers to identify physical reasons for possible inconsistencies, particularly related to sampling and measurement techniques. The continuous radon flux monitoring capability was analyzed to harmonize the radon flux methods under field conditions.

The results of these two intercomparison campaigns are presented and discussed in this paper. In order to facilitate the readability, the abbreviations and nomenclature used in the present manuscript has been compiled in Table 1.

## 2. Materials and Methods

### 2.1. Participants and Measurement Systems

The participant institutions in this exercise were the University of Cantabria, Spain (UC), the Universitat Politècnica de Catalunya, Spain (UPC), the Agenzia nazionale per le nuove tecnologie, l’energia e lo sviluppo economico sostenibile, Italy (ENEA), and the Horia Hulubei National Institute of Physics and Nuclear Engineering, Romania (IFIN-HH). Every institution participated with their own device, managed by themselves, except for UPC, which managed its own system and also an ANSTO Autoflux designed by the Australian Nuclear Science and Technology. All the systems involved in this study were made by a commercial continuous radon monitor (with or without pump) coupled with an accumulation chamber of different volume and shape, where the increase in the radon activity concentration over time was measured for calculating the radon flux, as it will be explained in detail later [16]. Table 2 summarizes the main characteristics of each system; the used acronym corresponds to the participant institution or designer.

- The ANSTO Autoflux system consists of an AlphaGUARD monitor (Bertin Instruments, Montigny-le-Bretonneux, France), a cylindrical drum, and several environmental sensors controlled by a Raspberry Pi. This system allows the retrieval of radon flux observations every three hours. The radon activity concentration within the drum accumulates during 1 h and then the drum is open during 2 h to be ventilated and to reduce the radon activity concentration within it.
- The system designed and built by UPC, called INTE_Flux, allows three radon flux measurements per day (each 8 h). It consists of a cylindrical metallic chamber, which can be opened/closed thanks to two electrovalves and a pump to help flush the air within the chamber. A DOSEman monitor (Sarad GmbH, Dresden, Germany) on diffusion mode measures the radon activity concentration within the chamber during 5 h; then, the valves open, and the pump is switched on to flush the chamber for 3 h.
- The UC system consists of a methacrylate box connected by two tubes to an RTM 2200 radon monitor (Sarad GmbH, Dresden, Germany). The accumulation chamber needs to be manually handled during monitoring to be ventilated.
- The ENEA system is made by a steel cylinder with an AlphaGUARD inside working in diffusion mode. This device has an upper passive valve that can be manually opened or closed during measurement. The role of the valve is under testing with the objective to continuously measure the radon flux from radon activity concentration variation inside the accumulation chamber.
- The IFIN-HH system consists of a Radon Scout monitor (Sarad GmbH, Dresden, Germany) measured in diffusion mode inside a cylinder of HDPE material. Similarly to the UC system, the IFIN-HH accumulation chamber needs to be opened manually due to the incapability to degas automatically.

### 2.2. Site Description

**Figure 1:**
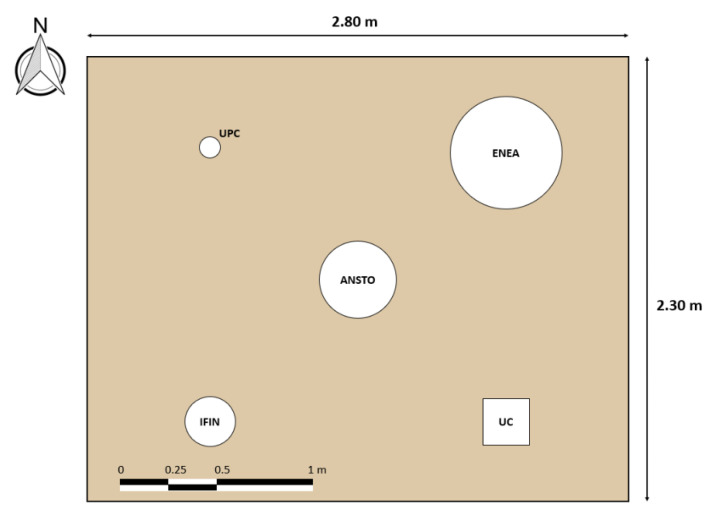
Position setup of the radon flux systems in the field. The shapes, positions, and dimensions are to scale.

The intercomparison campaigns were performed in two different fields: a low and a high radium activity area. Sites were selected on the basin of their radium content in soil with the purpose of studying the response and behavior of the systems both in areas where low and high radon exhalation rates were expected. The procedure followed to place the devices in the soil surface was the same for both experiments. First, the grass layer was removed in an area of dimensions 2.80 m × 2.30 m to simplify the installation of the accumulation chambers in the ground. The relative position of every device was the same in both campaigns, as shown in Figure 1. For each campaign, five soil samples were collected (at 4 corners and in the center of the area) in order to determine the average radium activity concentration (Bq/kg) of the field and the homogeneity. The samples were measured by the Laboratory of Environmental Radioactivity, University of Cantabria (LaRUC), accredited according to UNE-EN ISO/IEC 17025:2017 for activity concentration measurements of soil by gamma spectrometry using a high-purity germanium detector.

**Figure 2:**
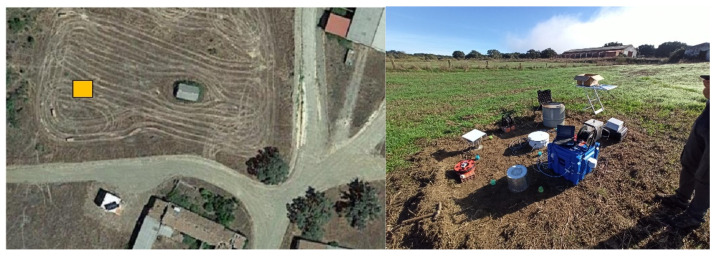
Aerial view and picture of the devices placed in the high radon flux area. The yellow rectangle indicates the testing area.

The site selected for the high radon flux campaign is within the land of a former uranium mine managed by the Spanish Uranium Company (ENUSA), located in Saelices el Chico (Salamanca, Spain) (latitude: 40.65, longitude: −6.63). The area chosen is called “Sageras”, and it is an undeveloped area near a pilot house built for radon remediation actions research [21,22]. The soil of the experimental area showed an average radium concentration of 814 ± 65 Bq/kg (k = 2). Figure 2 shows an aerial view of the selected area and displaced monitors during the experiment.

**Figure 3:**
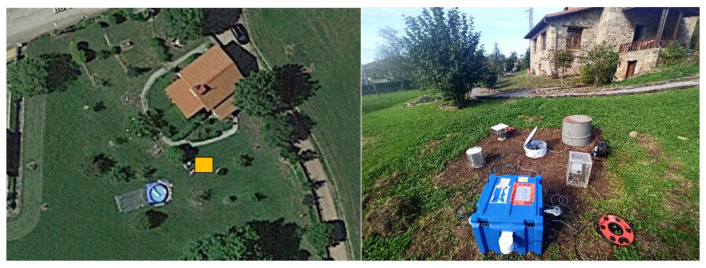
Aerial view and picture of the devices placed in the low radon flux area. The yellow rectangle indicates the testing area.

The selected low radon flux area is a private house garden located in Esles de Cayón (Cantabria, Spain) (latitude: 43.28, longitude: –3.80), with an average radium concentration average of (29 ± 3) Bq/kg (k = 2). The low radon flux campaign was conducted between 13 and 28 October 2021. Figure 3 shows an aerial view of this area and monitors during the experiment.

**Figure 4:**
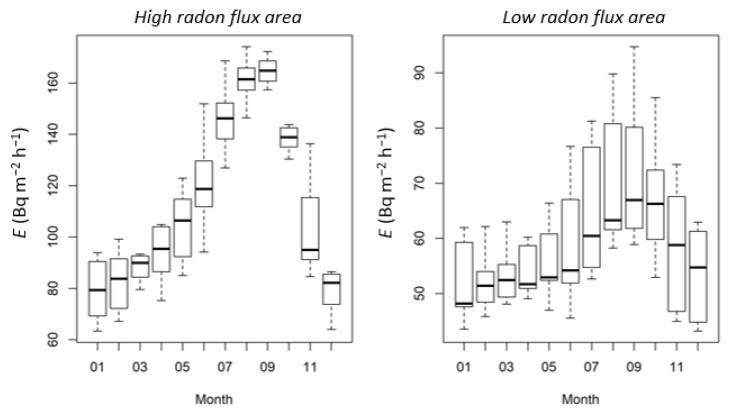
Radon flux monthly prediction obtained from the model proposed by Kartsten et al. 2015 [7] for each area chosen for the intercomparison radon flux campaigns.

Additionally, Figure 4 shows a boxplot of the monthly average of the radon flux predicted for these sites by the radon flux model presented in Karstens et al. 2015 using data for the period 2006–2010 based on soil moisture data from the Noah Land Surface Model in the Global Land Data Assimilation System (GLDAS Noah) [7]. The average monthly values of this model output for October climatology was about 138 Bq m^−2^ h^−1^ and 66 Bq m^−2^ h^−1^ at the high and at the low radium content areas, respectively.

Karstens et al. 2015 successfully compared this model with another available radon flux model [23], also based on radon transport equation in soil, and punctual radon flux measurements in Europe. The model, as declared by authors, calculates the radon source term proportionally to the ^226^Ra content of the soil. This is derived thanks to ^238^U values given in the Geochemical Atlas [24] and the conversion factor from ^238^U concentration to ^238^U activity concentration [25], i.e., 12.35 Bq kg^−1^ per mg kg^−1^ uranium. The radium concentration values extracted for the intercomparison areas were about 50 Bq/kg for the high radon flux area and of 35 Bq/kg for the low radon flux area.

For the high flux radon area, there is a great difference between the radium content in soil measured experimentally (814 Bq/kg) and the value used in the model (50 Bq/kg). Thus, considering the proportionality between the radium source term and the radon exhalation used into the model (138 ± 6 Bq m^−2^ h^−1^), the corrected radon flux from the model should be equal to 2247 ± 81 Bq m^−2^ h^−1^. Likewise, the radon flux proposed for the low radon flux area (66 ± 12 Bq m^−2^ h^−1^) has been corrected considering the radium content in soil measured experimentally (29 Bq/kg) in comparison with the value used by the model (35 Bq/kg). Therefore, the corrected radon flux value in this case is 56 ± 10 Bq m^−2^ h^−1^.

### 2.3. Radon Flux Calculation

The variation of the radon activity concentration *C* with the time *t* in the accumulation chamber can be modeled according to the differential equation [26,27]:

(1) $$\frac{dC\left(t\right)}{dt}=\frac{\varphi }{V}-\lambda \cdot C\left(t\right)$$

where

φ (Bq h^−1^): radon in soil production per unit of time;
*V* (m^3^): accumulation chamber volume;
$\lambda^{\;}$(h^−1^): effective decay constant. Sum of the removal constants: $\lambda =\lambda_{Rn}+\lambda_{b}+\lambda_{l}$, radon decay $\lambda_{Rn}$ + backdiffusion $\lambda_{b}$ + possible leaks of the system $\lambda_{l}$.

The solution of differential Equation (1) gives the radon concentration variation with time inside the chamber, which has an exponential behavior. Then, exhalation rate or radon flux *E* can be obtained from the parameters given by exponential adjustment of Equation (1)’s solution:

(2) $$C_{Rn}\left(t\right)=C_{0}e^{-\lambda t}+\frac{E}{V/S\cdot \lambda }\left(1-e^{-\lambda t}\right)$$

where

*C*_0_: initial radon concentration (*t* = 0);
*S*: Exhaling soil surface.

Considering that the background radon concentration in the chamber *C*_0_ is close to zero at the beginning of the accumulation process, the initial slope of the curve is independent of the backdiffusion [28]. Assuming that initially, the loss of radon through leaks is negligible ($\lambda \approx \lambda_{Rn}$) and that λ·t≪1, the exponential term can be approximated by $e^{-\lambda t}\approx 1+\lambda \cdot t$ according to Taylor series expansion. Therefore, the accumulation phase initially describes a linear growth of the radon concentration in the accumulation chamber described by:

(3) $$E=\frac{V}{S}\frac{C_{Rn}}{t}=h\frac{C_{Rn}}{t}$$

where *h* is the effective height, i.e., the ratio between *V*, the effective accumulation chamber volume (volume of the system where the sampled air can circulate), and *S*, the exhaling soil surface within the accumulation chamber.

**Figure 5:**
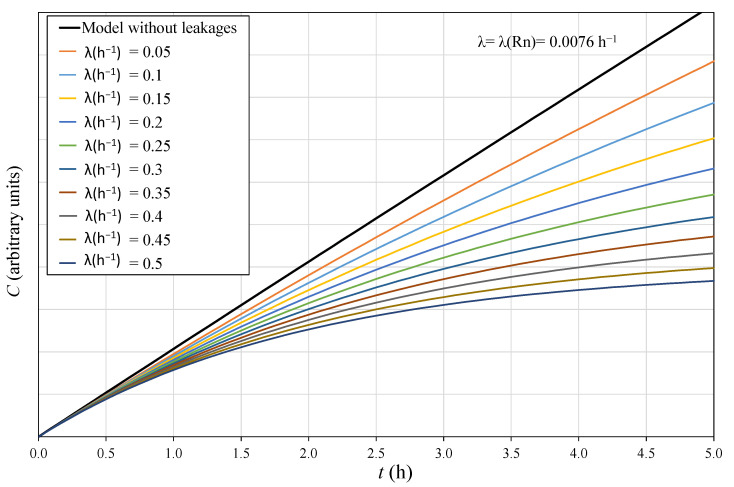
Theoretical approach of radon concentration over time for the first 5 h depending on λ (h^−1^) for the same radon flux value. Curves obtained from Equation (2) with a time interval of 10 min.

The linear behavior given by Equation (3) depends on the value of the effective decay constant and the time considered. Figure 5 shows the theoretical increase in the radon activity concentration in a given volume *V* over 5 h, based on Equation (2) for several λ values. It is observed that when there are no leakages, $\lambda \approx \lambda_{Rn}$, the behaviour of radon concentration is linear over the 5 h. However, for $\lambda >\lambda_{Rn}$, the trend of the radon concentration diverges from the linear behavior with time, in a way that, proportionally to the increase in λ, the linear period of the curves decreases. To minimize the error made in assuming the linear behavior of radon concentration in a given volume, it is necessary to check when linearity is satisfied depending on the two mentioned parameters λ and t.

This linear approach is useful to perform fast radon flux measurements needed to validate high temporal resolution models. As explained above, the linear behavior depends on the effective decay constant λ and the time considered t. Thus, for a given system, to evaluate the maximum measurement period that satisfies the linear behavior, the following should be considered: the λ of the system and the time frequency response of the radon monitor used, to ensure enough data to reduce the measurement uncertainty, not forgetting that λ could be variable over the measurement period due to environmental effects.

**Figure 6:**
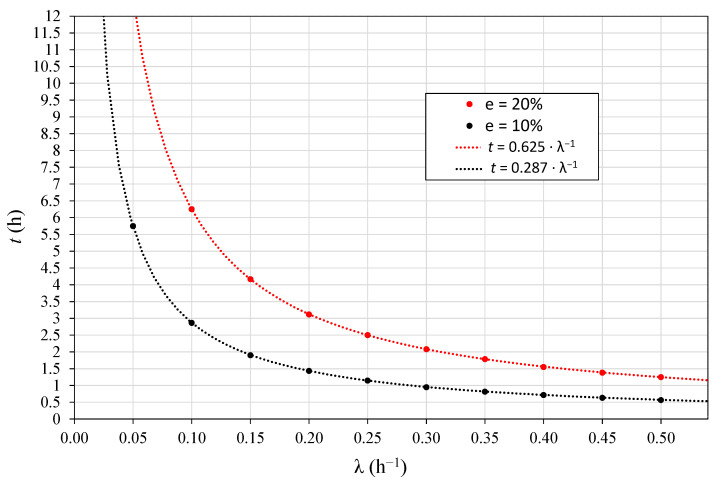
Theoretical estimation of time to be considered in the radon flux linear fitting calculation to not exceed a difference of 10% and 20%.

The radon exhalation value used for the theoretical approach in Figure 5 is the same for all the curves; the only difference between them is the effective decay constant. Considering different time periods, radon flux can be calculated from the curves using Equation (3). Then, the error due to the linear approximation can be obtained as the difference between the radon flux calculated assuming linearity and the real value. Maximum linearity time *t* can be estimated as a factor of λ for a fixed level of maximum deviation; 10% and 20% are considered here (see Figure 6). Given that, a relationship $t=a\cdot \lambda^{-1}$ is obtained with a  = 0.287 for 10% and a  = 0.625 for 20% maximum deviation. A similar theoretical study was completed [18,19] considering two points for the linear fit, but in this approach, pairs of values every 10 min from Figure 5 were used.

To check the applicability of the linear approach in the intercomparison campaigns, a static accumulation experiment is conducted at the beginning of the measurement to determine the effective decay constant λ of each system from the exponential adjustment of Equation (2). Considering the experimental uncertainties of radon measurements, we established an acceptable deviation of 20% to theoretically calculate the maximum linearity time.

From the data of the static accumulation measurement, radon flux is obtained both by using Equation (2) and also by the linear fit to Equation (3) using several time intervals for the first 5 h; then, these values are compared. Afterwards, taking into account the theoretical approach shown in Figure 6 and the type of device and its characteristics (integration time, radon monitor response, etc.), it is decided how much time should be considered for the linear fit in the next accumulation periods.

**Figure 7:**
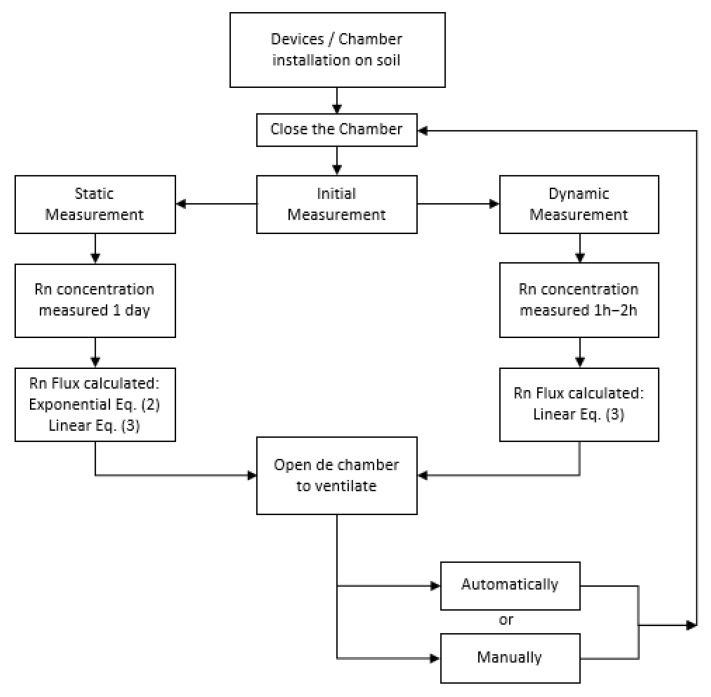
Flowchart of the methodology applied to radon flux measurements in field.

The static measurement experiment (24 h duration) intends to serve as reference to determine the leakage from the exponential adjustment, but before, it has to be considered that immediately after the installation of the chamber into the soil, some time should be allowed for the exhalation conditions to reach equilibrium. After the static measurement, the dynamic measurement commences; a flowchart explaining the measurement procedure followed for the campaigns is presented in Figure 7.

### 2.4. Results Assessment

The reference radon flux value for each area is obtained by consensus applying the iterative algorithm A, according to ISO 13528:2015 [29]. This algorithm is described and applied in other international interlaboratory exercises [20,30].

The declared radon flux result for each participant is the average of every measurement, and the parameters used to evaluate and compare them are the relative percentage difference *D*(%) and the z’-score (*z*’) [31,32,33]:

(4) $$D\left(\%\right)=100\frac{E_{i}-E_{ref}}{E_{ref}}$$

(5) $$z^{\prime }=\frac{E_{i}-E_{ref}}{\sigma }$$

where *E_i_* is the radon flux mean obtained by every participant and *E_ref_* is the flux reference value obtained according to ISO 13528:2015. *σ* is the standard deviation of the proficiency test established as 20% of the reference value for this study. Acceptance criteria of *z*′-score parameter is established as:

- *z*′ ≤ 2   Result satisfactory;
- 2 < *z*′< 3 Result questionable;
- *z*′ ≥ 3   Result not acceptable.

## 3. Results

This section describes the experimental results of the intercomparison campaigns both at the high and at the low radium activity areas. The procedure followed was the same for the two campaigns, as explained in the previous section (see flow chart in Figure 7). During the first day, all the devices were set to accumulate radon in a static mode: for the ANSTO, the drum lid was closed permanently, for the UPC device, the air pump was switched off and the valves were closed, and in the ENEA device, the upper valve was closed. The UC and IFIN-HH devices measure in a static way by default.

After the 24 h static accumulation experiment of the first day, all accumulation chambers were opened until the radon monitors showed background values. From this point, the accumulation chambers were opened and closed automatically (ANSTO and UPC), manually (UC and IFIN), and in the case of ENEA’s device, the upper valve was opened. The different phases of the measurement are shown in the following plots, which are indicated as follows:

- From point A to B: first static accumulation period (24 h).
- From point C: continuous mode for ANSTO and UPC is ON; opening of ENEA’s device upper valve.
- Red arrow: manual closing of UC and IFIN-HH accumulation chambers after the previous opening and degassing period.
- Orange arrow: start of natural accumulation chamber degassing event.

The radon concentration time series recorded by the radon monitors for the two in-tercomparison campaigns can be found in Supplementary Materials.

### 3.1. High Radon Flux Area Campaign (6–8 October 2021)

**Figure 8:**
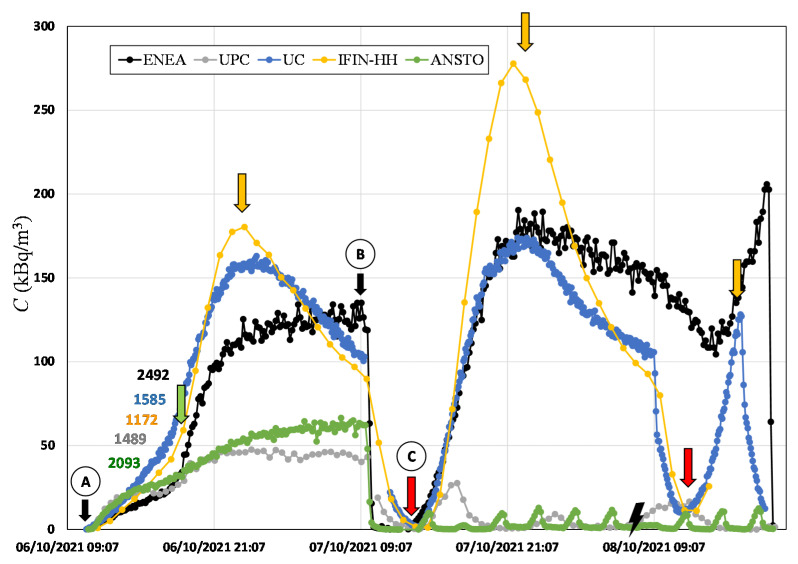
Radon activity concentration over time measured during the high radon flux campaign. Colored numbers indicate the radon flux (Bq m^−2^ h^−1^) calculated by linear fit for each corresponding system. From point A to point B is the static accumulation period. From point C starts the continuous measurement period.

The radon concentration time series measured by each monitor within their respective accumulation chambers during the high radon flux area campaign, carried out between 6 and 8 October 2021, is shown in Figure 8. The first accumulation period (point A to B, Figure 8) started on 6 October at 10:30 a.m. and lasted for approximately one day. A change in the trend of the radon concentration accumulated inside the chambers was observed after 6 h from the beginning of the measurement (green arrow in Figure 8). ANSTO, UPC, and ENEA devices describe two exponential growths. However, UC and IFIN-HH devices follow a sigmoidal curve, which was not the expected exponential behavior according to Equation (2). Furthermore, they experienced a degassing event at midnight (orange arrow in Figure 8). This could be explained due to the variation of the radon flux over the night and/or of the leakages of the system during the measurement period forced by environmental conditions in the soil (i.e., soil water content) or in the atmospheric layer in contact with the soil (temperature, pressure, wind, etc.).

The radon flux and effective decay constant for each system (see Table 3) were calculated only using the data observed during the first exponential growth (see Figure 8) to avoid the not justified behavior encountered after. For UC and IFIN-HH devices, it was not possible to calculate such parameters. The maximum linearity time calculated to obtain an error <20% was 2 h, 1 h, and 6 h for ANSTO, UPC, and ENEA devices, respectively. It is important to highlight that the first two data points (20 min) of the ANSTO Autoflux were not considered in the analysis because this instrument includes a 6 L delay volume to avoid thoron contribution, which influences the temporal response of the AlphaGUARD during the accumulation phase.

From point C of Figure 8, ANSTO Autoflux (ANSTO) and INTE_flux (UPC) systems started to measure automatically, providing 10 and three radon flux observations per day, respectively. Due to an electricity blackout event (ray symbol in Figure 8), a radon flux measurement was lost for these two systems. The ENEA system had the upper valve opened, and for UC and IFIN-HH systems, two radon flux measurements were performed by manually opening of their chambers (indicated by red arrows in Figure 8). Again, degassing events were observed for ENEA, UC, and IFIN-HH devices (orange arrows, Figure 8), which were caused probably by the temperature and pressure differences between inside and outside of the accumulation chambers. The ENEA system experienced this effect too, which was probably due to the presence of the open valve, which facilitates the escape of radon.

**Figure 9:**
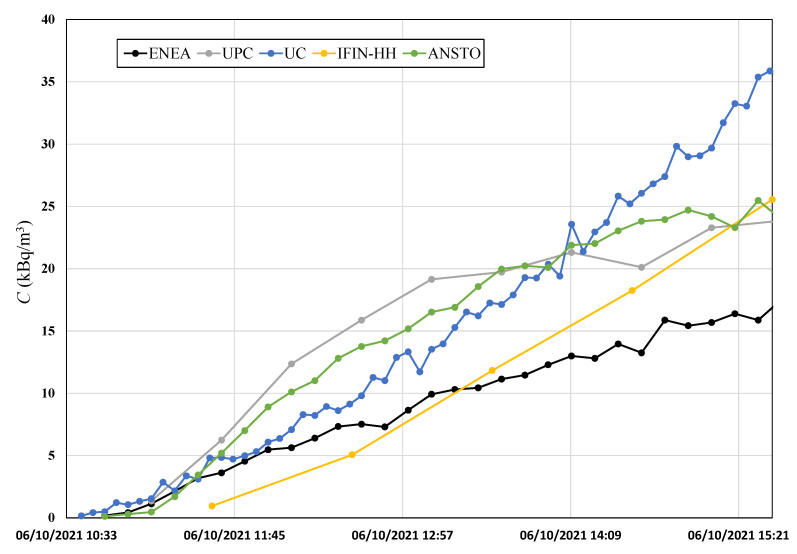
Radon activity concentration over the first 5 h of the static accumulation period during the high radon flux area campaign (from point A of Figure 8).

Figure 9 shows the first 5 h of measurement during the static accumulation experiment. Data from this experiment were used to obtain the radon flux for each system with different time intervals, and they are presented in Table 4. Comparing the radon flux results obtained from the linear fit and the exponential fit, and considering the theoretical approach of Figure 6, it was determined that the recommended time for the linear method application is 1 h for ANSTO, 2 h for UPC, and 1–2 h for ENEA, which ensures an agreement between both linear and exponential approaches of 2%, 4%, and 7%, respectively. In case of UC and IFIN-HH systems, the recommended time is 1 h and 3 h, respectively. The time considered for all devices is based on the previous results discussed; however, it is important to take into account the sensitivity and time response of the monitors.

**Figure 10:**
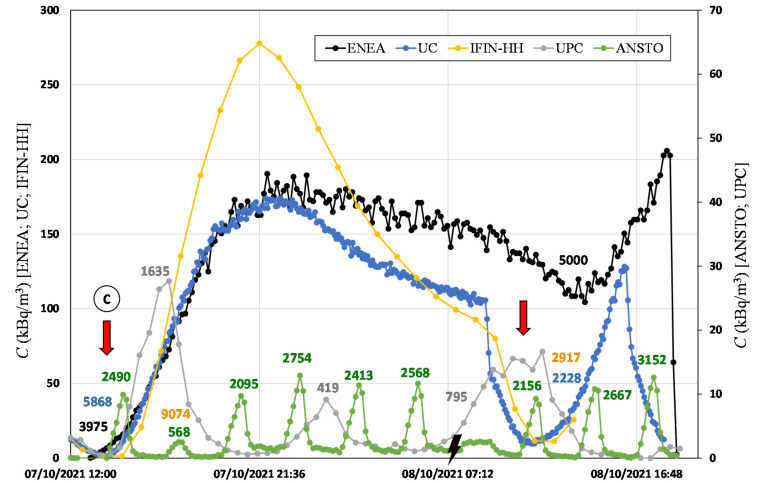
Radon activity concentration over time measured after the static accumulation period. Number labels indicate the radon flux (Bq m^−2^ h^−1^) calculated by linear fitting.

Figure 10 shows the second part of the intercomparison campaign performed at the high radium content area; labels indicate the radon flux calculated by each system over time. Table 5 presents for each system the mean radon flux value from the observations, its standard deviation over the population, the number of observations, and the time used for the application of the linear fit method.

Results between participants show significant differences, and some systems present a standard deviation higher than 50% of the corresponding mean value. These disagreements could be explained by the influence of environmental parameters on radon flux systems leakage during field measurements, increasing of the system leakage due to the concentration gradient between inside and outside the accumulation chambers, and respective temperature/pressure gradients. According to the participants’ experience in this type of high radon exhalation areas, the heterogeneity could contribute to the dispersion of the results [34].

### 3.2. Low Radon Flux Area Campaign (13–28 October 2021)

**Figure 11:**
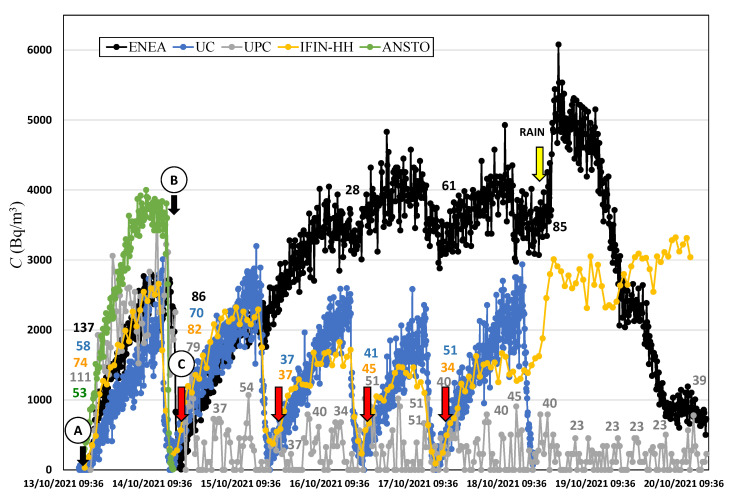
Radon activity concentration over time measured during the low radon flux campaign first week. Colored numbers indicate the radon flux (Bq m^−2^ h^−1^) calculated by linear fit for each corresponding system.

**Figure 12:**
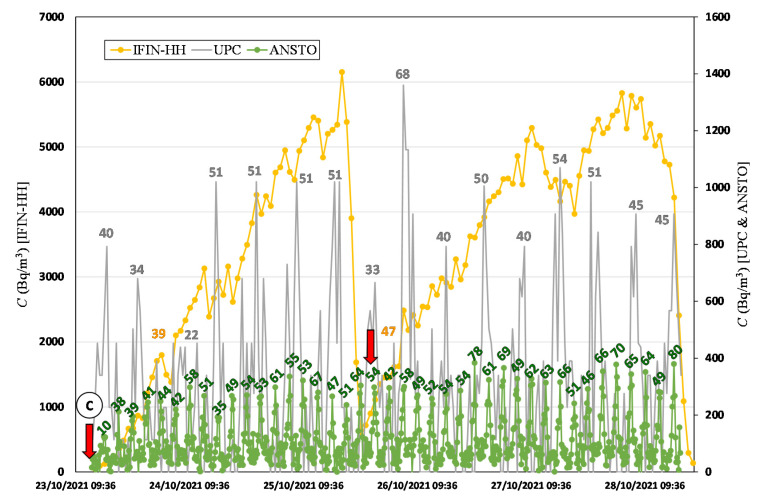
Radon activity concentration over time measured during the second week of the low radon flux area campaign. Colored numbers indicate the radon flux (Bq m^−2^ h^−1^) calculated by linear fitting.

The intercomparison campaign at the low radon flux area was divided in two periods, 13–20 October 2021 and 23–28 October 2021. The time series of radon activity concentration measured by each device is shown in Figure 11 and Figure 12. The initial static accumulation measurement, with all accumulation chambers closed (from point A to B in Figure 11), was performed from 13 October at 16:30 and lasted one day, approximately. Then, the continuous dynamic measurement period started, automatically for ANSTO and UPC (point C in Figure 11) and by manually opening/closing of UC and IFIN-HH accumulation chambers, and for the ENEA system, the upper valve was left open. Due to a setting error, the ANSTO Autoflux did not start to measure in the first period of the campaign, but it provided 41 radon flux observations during the second period. The UPC system provided 35 radon flux observations over the two periods. For UC and IFIN-HH systems, four and six radon flux measurements were manually performed, respectively. The ENEA device had one accumulation period after the initial static accumulation with three variations due to the opened valve. The partial increases during the accumulation were used to calculate the radon flux values for assessing if the open valve could be used for continuous measurements. For the second period of the campaign, only ANSTO, UPC, and IFIN-HH devices were installed.

During the last 2 days of the first campaign period, it started to rain (yellow arrow, Figure 11). Radon flux measured by the UPC system was reduced to almost 50%, and the radon activity concentration recorded by the ENEA monitor decreased drastically. However, the radon activity concentration measured within the IFIN-HH system increased. These two last systems could not be used to calculate the radon flux in this period.

From the first exponential growth, the radon flux and effective decay constant λ were obtained (see Table 6). In this case, the radon activity concentrations measured by each monitor within their respective accumulation chambers followed an exponential increase as described by Equation (2); thus, it was possible to evaluate the mentioned parameters following the theory. The maximum radon concentration values recorded were below 4 kBq/m^3^ in the low radon flux area, while over 150 kBq/m^3^ was recorded by some devices in the high radon flux area. The experimental leakages calculated for all systems during the static experiment were in this case lower than in the high radium content area, reinforcing the linearity behavior theory (Table 3). This fact could indicate that advection processes were probably much smaller here.

The results of radon flux obtained for each system using the linear approach in Equation (3), for different time intervals, are presented in Table 7. Again, comparing the radon fluxes obtained from the linear fit and the exponential fit, and taking into account the theoretical approach in Figure 6, the recommended time *T* to be used without underestimating the flux by over 20% is determined. This could be increased by 2–3 h.

Looking at the data of the DOSEman (UPC) during the low radon flux area campaign, it is only possible to define an interval for the radon flux values observed with this system because the monitor is not sensitive enough to small radon concentration variations and the diffusion mode does not help follow the fast radon concentration variations during the accumulation phase. The low sensitivity and slow response of the DOSEman (30 min) complicates the correct analysis of each slope. Thus, to calculate the radon exhalation, we only considered the difference between the maximum and minimum values measured during the accumulation phases.

Considering the radon flux calculated in all the accumulation periods for each device using the linear assumption, as indicated by the number labels in Figure 11 and Figure 12, the mean radon flux value and the standard deviation are obtained (see Table 8).

Results obtained at the low radon exhalation area between participant systems show a good agreement considering their standard deviations. It should be also noted that λ values calculated at this area were 50% lower than the ones calculated at the high radon flux area for the same devices and placement methodology. The radon activity concentrations measured inside the accumulation chambers were lower at this area, and that could lead to a smaller influence of environmental parameters. The experimental results are also in agreement with the radon flux values calculated for October month using the Kartens et al. (2015) model.

### 3.3. Summary of Results

Table 9 presents the reference value for radon flux and standard deviation obtained by consensus considering every single measurement given by participants in the high and low radon flux areas under study and the radon flux calculated at these areas using the output from Karstens et al. (2015). Results from this previous model were also corrected considering the experimental ^226^Ra content measured for the experimental sites. This reference value should not be taken as absolute, because it could be biased by the different number of observations given by each device. For instance, ENEA provides three and five results for each campaign, whereas ANSTO gives 10 and 42 results to the high and low campaigns, respectively. This consensus to estimate the reference value was chosen to reflect the total scatter of the robust mean given by SD*_ref_*. Table 9 also includes the radon flux prediction, average, and standard deviation for October month climatology from the model proposed by Kartsens et al. (2015) using directly the ^226^Ra used into the model and the experimental one. In case of the high radon flux area, the output increases from 138 to 2247 Bq m^−2^ h^−1^ when the measured radium content was considered. This value is consistent with the experimental radon flux observations in this area. A good agreement is observed between the experimental results and the output of the model at the low radium content area both for the measured and Geochemical Atlas radium data.

**Figure 13:**
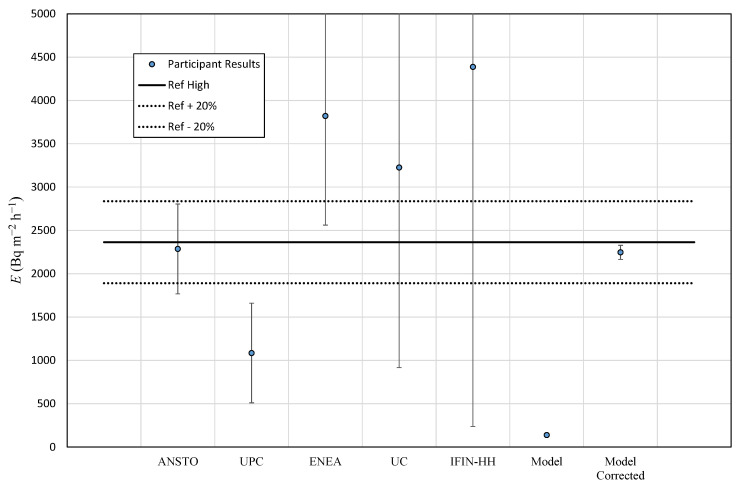
Participants results: average and reference value for the high radon flux area campaign.

**Figure 14:**
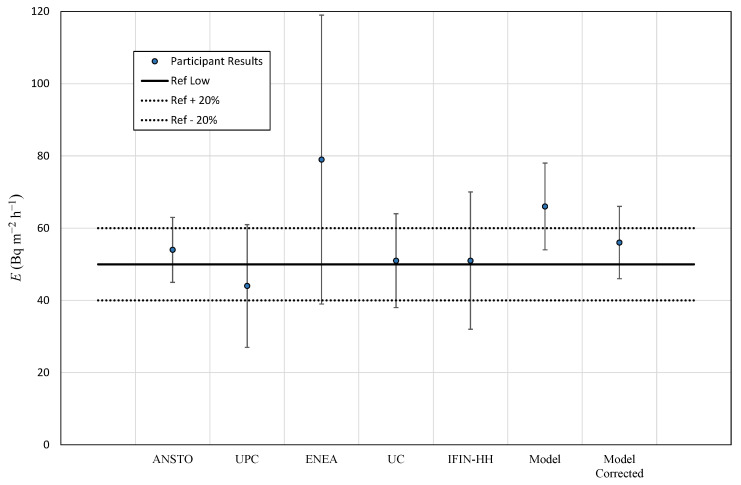
Participants results: average and reference value for the low radon flux area campaign.

Figure 13 and Figure 14 present the participants’ radon exhalation results with its standard deviation, obtained from Table 5 and Table 8, for the high and low radon area campaigns together with the model results. Additionally, the consensus value and an interval of ±20% from Table 9 are included. From the previous data, the relative difference and the *z*′-score were calculated; see Table 10. It is observed again the spread of the results for the high radon area campaign with great differences with consensus value, but the results are coherent considering the standard deviation. In case of low radon flux area campaign, *D* (%) is reduced in most of the cases, and the *z*′-score provides the most acceptable values compared to those in the high radon area campaign. Once again, the main difficulty of analyzing the results in a rigorous manner is the low number of results. The parameters of Table 10 can be taken as information, but the main affecting factor is the number of measurements.

## 4. Conclusions

Two intercomparison exercises with five different radon flux systems were conducted in October 2021 in the framework of the traceRadon project at low and at high radium content areas of Spain.

A methodology was proposed and followed to evaluate the maximum time interval to be used for each system in order to correctly calculate radon fluxes using a linear approach. This methodology is based on the evaluation of the system leakage by performing a 24 h accumulation experiment before starting continuous measurements. The introduction of this methodology in radon flux measurement protocols could be useful for obtaining high-frequency radon flux data with minimum possible uncertainties.

Radon flux results obtained at the high radium content area by the different systems under study show significant differences among them. In addition, there is a large spread of radon flux values for some devices considering the individual measurements. The dispersion of the results may be explained by large and variable leaks observed in the systems and possible radon flux variability over time. Only a few data were analyzed due to the short duration of the campaign, so robust statistical analysis was not possible. The radon flux reference obtained by consensus may include a potential bias induced by each device’s different number of measurements. However, the mean radon flux obtained in this area from the experimental observations was coherent with the value calculated using the Karstens et al. (2015) model after correcting the radium in soil content with the experimental one.

Radon flux results observed at the low radium content area provide interesting outcomes for common soils, usually presenting a similar average radium content. The results given by the different systems participating in the low radon flux area campaign are coherent among them and agree with the model prediction from Karstens et al. (2015) at this site. The results seem to indicate that radon fluxes lower than 100 Bq m^−2^ h^−1^ should be measured with high sensitivity and high response time monitors to reduce the uncertainty of short-term measurements. Another option could be increasing the time considered in the linear fitting, which is possible in the case that leakages calculated by a 24 h experiment are small.

The results of the intercomparison campaigns indicate that the radon concentration evolution in the accumulation chambers is limited by the installation of the system, the radon monitors characteristics (diffusion or pump mode, integration time, sensitivity, etc.), and the features of the accumulation chamber (material, volume, tubes, etc.), although it was not studied in detail here. The changes in the environmental conditions during the measurement also play a key role in the measurement and should be further investigated.

Overall, different radon flux systems were tested in the field under different radium content conditions. The work conducted contributes to the development of guidelines and a protocol for the harmonization of radon flux measurements in the field.

## Figures and Tables

**Table 1:** Abbreviations and nomenclature.

Abbreviation	Nomenclature
ANSTO	Australian Nuclear Science and Technology
BSS	Basic Safety Standards
EMPIR	European Metrology Programme for Innovation and Research
ENEA	Agenzia nazionale per le nuove tecnologie, l’energia e lo sviluppo economico sostenibile
ENUSA	Spanish Uranium Company
GHGs	Greenhouse Gases
GLDAS	Global Land Data Assimilation System
HDPE	High Density Polyethylene
IEC	International Electrotechnical Commission
IFIN-HH	Horia Hulubei National Institute for R&D in Physics and Nuclear Engineering
INTE	Institute of Energy Technologies
ISO	International Organization for Standardization
LaRUC	Laboratory of Environmental Radioactivity, University of Cantabria
RPA	Radon Priority Areas
RTM	Radon Tracer Method
UC	University of Cantabria
UPC	Universitat Politècnica de Catalunya

**Table 2:** Device features used by every participant.

Acronym	Radon Monitor				Accumulation Chamber			
	Device	Integration Time (min)	Sensitivity ^1^ (cpm/kBq/m^3^)	Mode	Diameter (cm)	Height (cm)	Shape	Material
ANSTO	AlphaGUARD	10	50	Pump	39	15	Cylinder	Steel
UPC	DOSEman	30	0.32	Diffusion	11	15	Cylinder	Steel
UC	RTM 2200	5	7	Pump	24 (side)	34	Square prism	PMMA
ENEA	AlphaGUARD	10	50	Diffusion	58	49	Cylinder	Steel
IFIN-HH	Radon Scout	60	1.8	Diffusion	26	24	Cylinder	HDPE

^1^ Declared by the manufacturer.

**Table 3:** Total leakage λ and radon flux *E* obtained from the exponential fit applied over a static accumulation experiment for ANSTO, UPC, and ENEA devices at the high radon flux area campaign.

Device	λ (h^−1^)	*E* (Bq m^−2^ h^−1^)
ANSTO	0.40	2130
UPC	0.74	1435
ENEA	0.10	2278

**Table 4:** Radon flux *E* (Bq m^−2^ h^−1^) obtained from the linear fit applied over a static accumulation experiment considering some time period *T* for each device.

*T* (h)	ANSTO	UPC	ENEA	UC	IFIN-HH
1	2093	-	2462	1585	-
2	1697	1489	1951	1707	886
3	1519	1149	1882	1999	1172
4	1348	829	1785	2233	1264
5	1139	686	1718	2459	1344

**Table 5:** Mean radon flux *E* obtained from the average of *n* number of measurements carried out in the high radon flux area campaign considering a time period *T* in the linear fit and the standard deviation SD of the mean.

Device	*E* (Bq m^−2^ h^−1^)	SD (Bq m^−2^ h^−1^)	*n*	*T* (h)
ANSTO	2287	519	10	1
UPC	1085	575	4	2
ENEA	3822	1261	3	1
UC	3227	2310	3	1
IFIN-HH	4388	4151	3	3

**Table 6:** Total leakages λ and radon flux *E* obtained from the exponential fit applied over a static accumulation experiment at the low radon flux area campaign.

Device	λ (h^−1^)	*E* (Bq m^−2^ h^−1^)
ANSTO	0.11	94
UPC	0.39	70
ENEA	0.23	106
UC	0.088	62
IFIN-HH	0.093	75

**Table 7:** Radon flux *E* (Bq m^−2^ h^−1^) obtained from the linear fit applied over a static accumulation experiment considering some time period *T* for each device.

*T* (h)	ANSTO	UPC	ENEA	UC	IFIN-HH
1	53	-	181	49	45
2	64	78	137	58	39
3	62	111	126	48	74
4	66	80	107	47	76
5	66	57	103	49	64

**Table 8:** Mean radon flux *E* obtained from the average of *n* number of measurements carried out in the low radon flux area campaign considering a time period *T* in the linear fit and the standard deviation SD of the mean.

Device	*E* (Bq m^−2^ h^−1^)	SD (Bq m^−2^ h^−1^)	*n*	*T* (h)
ANSTO	54	9	42	1
UPC	44	17	36	3
ENEA	79	40	5	2
UC	51	13	5	2
IFIN-HH	51	19	7	3

**Table 9:** Reference radon flux value *E_ref_* and standard deviation SD*_ref_* obtained from participant results according to ISO 13528:2015, including the parameter σ = 0.2· *E_ref_*. “Model” indicates the prediction proposed by Kartsens et al. (2015) for the month of October and “Model Corrected” indicates the prediction adjusted considering the experimental values measured for radium in soil content. Values are expressed in Bq m^−2^ h^−1^.

Area	*E_ref_*	SD*_ref_*	*σ*	Model	Model Corrected
High	2364	1172	473	138 ± 6	2247 ± 81
Low	50	15	10	66 ± 12	56 ± 10

**Table 10:** Relative difference percentage *D* (%) and *z*′-score calculated from participant results and the consensus value.

Device	High Campaign		Low Campaign	
	*D* (%) (Bq m^−2^ h^−1^)	*z*′-Score	*D* (%) (Bq m^−2^ h^−1^)	*z*′-Score
ANSTO	−3	0.1	8	0.4
UPC	−54	2.7	−12	0.6
ENEA	62	3.1	58	2.9
UC	37	1.8	2	0.1
IFIN-HH	86	4.3	2	0.1

## Data Availability

The data presented in this study are available in supplementary material.

## Supplementary Materials

The following supporting information can be downloaded at: https://www.mdpi.com/article/10.3390/ijerph19074213/s1, File S1: Data series of radon activity concentration.
